# The Effect of Continuous Ventilation on Thiol-Disulphide Homeostasis and Albumin-Adjusted Ischemia-Modified Albumin During Cardiopulmonary Bypass

**DOI:** 10.21470/1678-9741-2018-0398

**Published:** 2019

**Authors:** Seyda Efsun Ozgunay, Kadir Kaan Ozsin, Yasemin Ustundag, Derya Karasu, Buket Ozyaprak, Burak Balcı, Ozcan Erel, Senol Yavuz

**Affiliations:** 1 Department of Anesthesiology, University of Health Sciences, Bursa Yuksek Ihtisas Training and Research Hospital, Bursa, Turkey.; 2 Department of Cardiovascular Surgery,University of Health Sciences, Bursa Yuksek Ihtisas Training and Research Hospital, Bursa, Turkey.; 3 Department of Medical Biochemistry, University of Health Sciences, Bursa Yuksek Ihtisas Training and Research Hospital, Bursa, Turkey.; 4 Department of Medical Biochemistry, University of Health Sciences, Ankara Dıskapi Yıldırım Beyazıt Research and Education Hospital, Ankara, Turkey.

**Keywords:** Coronary Artery Bypass, Mechanical Ventilation, Ischemia-Modified Albumin, Disulfides, Sulfhydryl Compounds, Biomarkers

## Abstract

**Objective:**

To investigate the effect of continuous lung ventilation with low tidal volume on oxidation parameters, such as thiol/disulphide homeostasis and albumin-adjusted ischemia-modified albumin (AAIMA), during cardiopulmonary bypass (CBP) in coronary artery bypass grafting (CABG).

**Methods:**

Seventy-four patients who underwent elective CABG with CPB were included in the study. Blood samples were taken in the preoperative period, 10 minutes after CPB, and six and 24 hours postoperatively. Patients were assigned to the continuous ventilation group (Group 1, n=37) and the non-ventilated group (Group 2, n=37). The clinical characteristics, thiol/disulphide homeostasis, ischemia-modified albumin (IMA), and AAIMA levels of the patients were compared.

**Results:**

A significant difference was found between the groups regarding native thiol, total thiol, and IMA levels at the postoperative 24^th^ hour (*P*=0.030, *P*=0.031, and *P*=0.004, respectively). There was no difference between the groups in terms of AAIMA. AAIMA levels returned to preoperative levels in Groups 1 and 2, at the 6^th^ and 24^th^ postoperative hours, respectively. Length of hospital stay was significantly shorter in Group 1 (*P*<0.001) than in Group 2.

**Conclusion:**

Continuous ventilation during CPB caused an increase in native and total thiol levels, an earlier return of AAIMA levels, and shorter hospital stay. Continuous ventilation may reduce the negative effects of CPB on myocardium (Table 2, Figure 1, and Reference 31).

**Table:** 

Abbreviations, acronyms & symbols				
AAIMA	= Albumin-adjusted ischemia-modified albumin		ICU	= Intensive care unit
ABSU	= Absorbance units		IMA	= Ischemia-modified albumin
ASA	= American Society of Anesthesiologists		I/R	= Ischemia/reperfusion
BSA	= Body surface area		MI	= Myocardial injury
CABG	= Coronary artery bypass grafting		PEEP	= Positive end expiratory pressure
CAD	= Coronary artery disease		SD	= Standard deviation
CPB	= Cardiopulmonary bypass		SH total SH	= Native thiol/total thiol percent ratio
EF	= Ejection fraction		SPSS	= Statistical Package for the Social Sciences
EuroSCORE	= European System for Cardiac Operative Risk Evaluation		SS total SH	= Disulfide/total thiol percent ratio
FiO_2_	= Fraction of inspired oxygen		SSSH	= Disulphide/native thiol ratio

## INTRODUCTION

Coronary artery bypass grafting (CABG) surgery is one of the major surgeries with high postoperative complications, mortality, and morbidity. Ischemia created during this process and following reperfusion can damage the myocardium. Inflammatory and systemic oxidative stress response caused by total cardiopulmonary bypass (CPB) causes endothelial damage in many systemic organs^[1-3]^, and this mechanism has been not fully explained yet. In addition, alveolar collapse, change in compliance of the chest wall, diaphragmatic dysfunction, lung and phrenic nerve trauma, splanchnic hypoperfusion, pulmonary ischemia/reperfusion (I/R), and use of protamine are also causes of the ischemic condition. Hemolysis in CABG activates neutrophils and the oxidative system and affects the myocardium, lungs, and kidneys by causing protein and lipid peroxidation and deoxyribonucleic acid oxidation, which affect clinical outcomes^[4]^.

Decreased myocardial blood flow causes the formation of ischemia-modified albumin (IMA) by causing hypoxia, acidosis, an increase in reactive oxygen derivatives, and change in serum albumin^[5]^. IMA, which reflects myocardial ischemia in minutes, shows the short-term oxidative effect^[6]^. The albumin-adjusted ischemia-modified albumin (AAIMA) level is also used because IMA levels may be affected by changes in the concentration of albumin^[7]^. Thiols are sulphur group-containing compounds, which are essential antioxidant buffers that interact with almost all physiologic oxidants^[8]^. Thiol groups are oxidized by oxidant molecules in the surroundings and converted to disulphide structures and then to thiol groups, thereby forming the equilibrium of thiol/disulphide. Thiol/disulphide homeostasis plays an important role in vital functions and antioxidant protection^[9]^.

Due to the fact that the heart is the most exposed organ to severe I/R, various studies have investigated the relationship between CABG, various CBP systems, surgical methods, anesthesia methods, administration of trace elements and vitamin supplements to patients, and the antioxidant status of patients^[1,2,10]^. Although there are many studies on continuous ventilation in CABG, randomized studies are limited^[11]^. In light of these studies, ventilation appears to be beneficial in the long term in total CPB. In the literature, no study has shown the effect of continuous ventilation during total CPB on thiol/disulphide homeostasis, IMA, and AAIMA levels.

It is known that continuous ventilation during CABG decreases the blood flow to the bronchial artery, reduces ischemia, and enables better inspiratory capacity by reducing lung damage^[12-15]^. In this study, we aimed to investigate the effect of continuous lung ventilation with low tidal volume on oxidation parameters, such as thiol/disulphide homeostasis, IMA, and AAIMA levels, during CBP in CABG.

## METHODS

### Study Design and Patient Selection

The study protocol was approved by the local Ethics Committee. Written informed consent was obtained from patients. The study was conducted in accordance with the principles of the Declaration of Helsinki. This prospective, randomized, single-center, double-blinded study was carried out between January 2018 and June 2018. The physician who evaluated the patients and their laboratory results were blinded to the study.

Patients with the American Society of Anesthesiologists (ASA) physical status III-IV who were aged 50-70 years and underwent elective CABG with CPB were included in the study. Smokers, patients who had not smoked for less than five years, patients with diabetes mellitus, with restrictive or obstructive pulmonary disease, with high pulmonary artery pressure in preoperative echocardiography and severe left ventricular failure, with ejection fraction (EF) <30% in echocardiography, with preoperative cerebrovascular disease, peripheral artery disease, renal insufficiency, patients receiving preoperative inotropic or mechanical support, patients who would undergo redo surgery, patients with inflammatory and rheumatic disease, and patients using corticosteroids and antioxidants were excluded from the study. The patients’ demographic data were recorded. Patients were randomly assigned to Group 1 (n=37), who were ventilated, and Group 2 (n=37), who were not ventilated, using the sealed envelope technique.

### Anesthesia Management

Standardized anesthesia management was performed according to the institutional standardized protocol by the same anesthesiologist. Patients were monitored and invasive catheterization to the radial artery was performed after intravenous midazolam (Zolamid®, Defarma, Tekirdag, Turkey) (0.05-0.1 mg/kg) was given. All patients were induced with fentanyl (Talinat®, Vem, Istanbul, Turkey) (1-2 µg/kg) and pentothal (Pental® Sodium, Istanbul, Turkey) (5-7 mg/kg); rocuronium bromide (Curon®, Mustafa Nevzat, Istanbul, Turkey) (0.6 mg/kg, intravenously) was used to assist endotracheal intubation. Sevoflurane (Sevorane®, Abbvie, Istanbul, Turkey) was used with 50% oxygen and 50% air mixture for maintenance of anesthesia. A Primus® (Draeger Medical, Lübeck, Germany) anesthetic machine was used for intraoperative mechanical ventilation. Surgery was started after internal jugular vein catheterization. During the operation, electrocardiography, arterial blood pressure, end-tidal carbon dioxide, pulse oximetry, temperature, and urine output were monitored. Intravenous fentanyl (3-5 µg/kg) was added before sternotomy. Heparin (300-400 units/kg) was administered to achieve a clotting time >480 seconds before cannulation. During total CPB, maintenance of anesthesia was achieved with midazolam, fentanyl, and rocuronium.

### Cardiopulmonary Bypass Management

Standard CPB was performed with mild hypothermia (32ºC). After median sternotomy and heparinization, CPB was performed with aorto-venous two-stage cannulation. A cross-clamp was placed to the ascending aorta and cardiac arrest was provided with cold antegrade cardioplegia with high potassium. Continuity of the cardiac arrest was provided with blood cardioplegia given every 15-20 minutes. CPB was established with a roller pump with a membrane oxygenator (Maquet, Getinge group, Restalt, Germany) and arterial line filter at pump flow rates of 2-2.4 L/min/m^2^. Arterial blood gas was analyzed every 20-30 minutes. Five hundred milliliters of hot blood cardioplegia was given just before the cross-clamp was removed.

### Ventilation Protocol

In Group 1, a ventilation strategy similar to the strategy used in the study of Durukan et al.^[16]^ was used during total CPB. Patients were ventilated with 5 mL/kg tidal volume, respiration rate, positive end expiratory pressure (PEEP) 0, and fraction of inspired oxygen (FiO_2_) (50% air, 50% oxygen) 0.5. In Group 2, the endotracheal tube was opened to room air during CPB and ventilation was discontinued. After the completion of the operation, all patients were taken to the intensive care unit (ICU) and standard postoperative care was performed. After the patients were awakened and hemodynamic stability was obtained, extubation was performed with the routine procedure at the earliest possible stage. Cross-clamp time, total bypass time, total anesthesia time, number of anastomosis, length of ICU stay, and hospital stay days were recorded.

### Blood Sampling

Blood samples were taken from all patients in the preoperative period (T0), at the 10^th^ minute after total CPB (T1), and at the postoperative 6^th^ (T2) and 24^th^ hours (T3). Blood samples were centrifuged at 3600 rpm for 10 min and serum plasma samples were removed and stored at -80ºC until analysis. Native thiol, total thiol, disulphide, disulphide/native thiol ratio (SSSH), disulphide/total thiol percent ratio (SS total SH), native thiol/total thiol percent ratio (SH total SH), and IMA levels were studied.

### Oxidation-Antioxidant Measurements

A new spectrophotometric technique used to establish thiol/disulphide homeostasis was previously described by Erel and Neselioglu^[17]^. IMA was studied using the spectrophotometric method defined by Bar-Or et al.^[18]^ and reported as absorption units. Albumin-adjusted IMA was calculated according to the formula=[(ındividual serum albumin concentration/median albumin concentration of the population)×IMA value].^[7]^

### Statistical Analysis

Statistical analyses were performed using the Statistical Package for the Social Sciences software (IBM SPSS Statistic Inc., Chicago, IL, USA), version 21.0. Continuous and ordinal variables were expressed as mean ± standard deviation and nominal variables were expressed as frequency and percentage. The normality of the continuous variables was analyzed with Kolmogorov-Smirnov test and Shapiro-Wilks test. Student’s t-test was used to compare two groups for continuous variables with normal distribution. Pearson’s chi-squared test was used to detect differences between groups on the basis of the categorical variables. Mann-Whitney U test was performed to compare two groups for continuous variables without normal distribution. To assess the relationship between measurements of mean serum albumin, IMA, AAIMA, native-thiol, total thiol, disulphide, SSSH, SS total SH, and SH total SH levels at different times, the paired *t*-test and the Wilcoxon signed-rank test were used in independent groups. A statistical significance was established when the *P*-value was <0.05.

## RESULTS

**Fig. 1:**
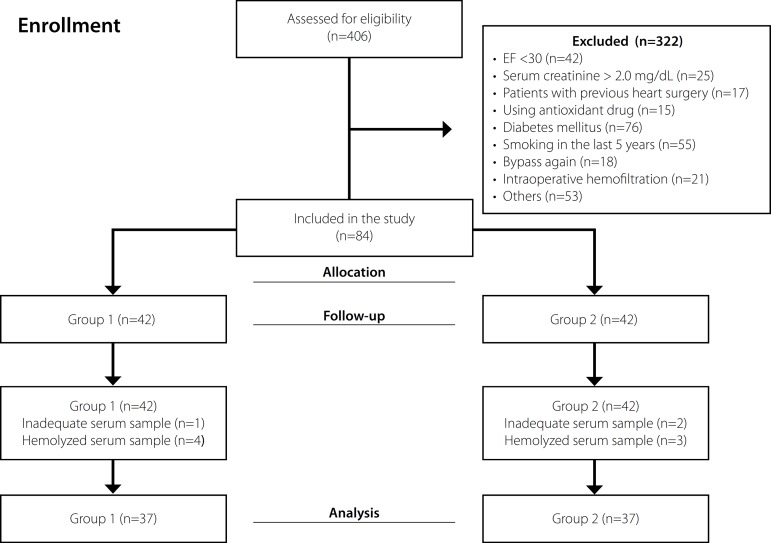
Flow chart of the study. EF=ejection fraction

Four hundred and six patients who underwent elective CABG were included in the study. Forty-two patients with EF <30%, 25 with serum creatinine >2.0 mg/dL, 17 with prior cardiac surgery, 15 using drugs that affected the antioxidant system, 76 with diabetes mellitus, 55 with a history of smoking in the last five years, 18 who were in need of undergoing bypass again, 21 who required intraoperative hemofiltration, and 53 for other reasons were excluded from the study. Eighty-four patients were included in the study; however, only 74 patients were included in the final statistical evaluation because the serum of seven patients was hemolyzed, and three patients’ serum samples were inadequate (Figure 1). No significant difference was detected regarding demographic data (*P*>0.05) (Table 1).

**Table 1:** Patients' demographic features.

Variable	Group 1 (n=37)	Group 2 (n=37)	*P*-value
Age, years (mean±SD)	61.14±9.49	61.11±9.03	0.990
Male gender, n (%)	28 (75.7)	32 (86.5)	0.187
ASA III/IV, n	16/21	13/24	0.317
EuroSCORE	4.41±1.77	4.46±1.77	0.903
BSA	1.89±0.15	1.89±0.19	0.834
Hypertension, n (%)	22 (59.5)	28 (75.7)	0.214
EF, % (mean±SD)	51.92±9.29	50.68±8.18	0.543
Cross-clamp time, min (mean±SD)	71.35±31.29	66.54±19.55	0.837
Cardiopulmonary bypass time, min (mean±SD)	99.49±37.26	96.19±26.27	0.846
Total anesthesia time, min (mean±SD)	269.54±54.63	247.57±39.72	0.052
Number of anastomoses	3.03±1.36	3.38±0.86	0.189
Number of erythrocytes	2.54±1.28	2.35±0.92	0.468
Number of plasma	3.84±1.17	3.76±1.28	0.776
ICU stay, days (mean±SD)	2.27±0.69	2.22±0.42	0.686
Hospital stay, days (mean±SD)	5.16±2.27	6.89±0.57	<0.001^#^

ASA=American Society of Anesthesiologists' physical status classification; BSA=body surface area; EF=ejection fraction; EuroSCORE=European System for Cardiac Operative Risk Evaluation; ICU=ıntensive care unit; SD=standard deviation

Group 1=Continuous ventilation group; Group 2=Nonventilated group;

# Mann-Whitney U test

In the comparisons between the groups, no differences were found in terms of T0, T1, and T2’s native thiol, total thiol, disulphide, SSSH, SS total SH, SH total SH, IMA, and AAIMA levels (*P*>0.05, Table 2). There were statistically significant differences between the groups in terms of native thiol, total thiol, and IMA levels at the postoperative 24^th^ hour (respectively, *P*=0.030, *P*=0.031, and *P*=0.004) (Table 2).

**Table 2:** Thiol/disulphide homeostasis, ischemia-modified albumin levels, and albumin-adjusted ischemia-modified albumin.

Parameters		T0	T1	T2	T3	*P*-value
Native thiol (µmol/L)	Group I (*n*=37)	181.23±48.33	84.14±44.55	202.31±49.98	245.24±57.45	T0-T1 <0.001¶ T0-T2 0.357 T0-T3 <0.001
	Group II (*n*=37)	178.78±41.06	99.67±50.24	194.94±43.73	219.44±41.52	T0-T1 <0.001¶ T0-T2 0.055 T0-T3 <0.001¶
	*P*	0.815	0.155	0.502	0.030*	
Total thiol (µmol/L)	Group I (*n*=37)	205.73±52.76	102.97±44.55	230.68±51.22	269.85±57.81	T0-T1 <0.001¶ T0-T2 0.027¶ T0-T3 <0.001¶
	Group II (*n*=37)	206.45±46.94	119.48 ±53.20	220.86±45.41	243.53±43.89	T0-T1 <0.001¶ T0-T2 0.099 T0-T3 <0.001¶
	*P*	0.951	0.178	0.386	0.031*	
Disulphide (µmol/L)	Group I (*n*=37)	12.75±6.13	9.41±4.44	14.18±5.47	12.31±3.69	T0-T1 0.012¶ T0-T2 0.210 T0-T3 0.662
	Group II (*n*=37)	13.82±4.91	9.90±3.44	12.96±5.27	11.91±4.15	T0-T1 <0.001¶ T0-T2 0.414 T0-T3 0.053
	*P*	0.410	0.599	0.331	0.664	
Disulphide/native thiol ratio ' 100	Group I (*n*=37)	7.62±4.80	15.48±12.96	7.80±4.87	5.38±2.29	T0-T1 <0.001¶ T0-T2 0.866 T0-T3 0.011¶
	Group II (*n*=37)	7.83±2.59	11.95±5.95	6.97±3.30	5.59±2.15	T0-T1 <0.001¶ T0-T2 0.217 T0-T3 <0.001¶
	*P*	0.317	0.140	0.492	0.492	
Disulphide/total thiol ratio ' 100	Group I (*n*=37)	6.33±3.27	10.54±5.90	6.48±3.17	4.77±1.79	T0-T1 <0.001¶ T0-T2 0.832 T0-T3 0.010¶
	Group II (*n*=37)	6.68±1.95	9.30±3.60	5.97±2.45	4.96±1.65	T0-T1 <0.001¶ T0-T2 0.180 T0-T3 <0.001¶
	*P*	0.302	0.338	0.520	0.641	
Native thiol/total thiol ratio ' 100	Group I (*n*=37)	87.47±6.49	78.76±11.85	87.04±6.34	90.45±3.59	T0-T1 <0.001¶ T0-T2 0.757 T0-T3 0.013¶
	Group II (*n*=37)	88.63±3.90	81.42±7.19	87.99±4.92	90.06±3.30	T0-T1 <0.001¶ T0-T2 0.194 T0-T3 <0.001¶
	*P*	0.504	0.246	0.470	0.446	
IMA (ABSU)	Group I (*n*=37)	0.69±0.13	1.07±0.14	0.77±0.15	0.77±0.12	T0-T1 <0.000¶ T0-T2 0.008¶ T0-T3 0.007¶
	Group II (*n*=37)	0.73±0.12	1.00±0.16	0.77±0.10	0.82±0.09	T0-T1 <0.001¶ T0-T2 0.089 T0-T3 <0.001¶
	*P*	0.052	0.112	0.961	0.004^#^	
Albumin	Group I (*n*=37)	3.28±0.38	1.41±0.72	2.96±0.77	3.01±0.10	T0-T1 <0.001µ T0-T2 <0.029µ T0-T3 0.014µ
	Group II (*n*=37)	3.1±0.57	1.81±1.00	3.10±0.42	2.68±0.82	T0-T1 <0.001µ T0-T2 0.698 T0-T3 0.013µ
	*P*	0.182	0.095	0.910	0.079	
AAIMA	Group I (*n*=37)	0.68±0.12	0.97±0.38	0.68±0.15	0.71±0.10	T0-T1 <0.001¶ T0-T2 0.897 T0-T3 0.412
	Group II (*n*=37)	0.69±0.08	1.15±1.51	0.73±0.06	0.68 ±0.17	T0-T1 <0.001¶ T0-T2 0.014¶ T0-T3 0.788
	*P*	0.204	0.143	0.336	0.534	

AAIMA=albumin-adjusted ischemia-modified albumin; ABSU=absorbance units; IMA=ıschemia-modified albumin

Data presented show values at the moment of T0=preoperatively; T1=at the 10th minute after total cardiopulmonary bypass; T2=postoperative 6th hour; and T3=postoperative 24th hour. Group 1=Continuous ventilation group, Group 2=Nonventilated group. A *P*-value of <0.05 is statistically significant.

# Mann-Whitney U test;

* Student's t-test;

µ Wilcoxon signed-rank test;

¶ Paired sample test

In the comparison of T0 and T1 between each group, there were statistically significant differences in terms of serum native thiol, total thiol, disulphide, SSSH, SS total SH, SH total SH, IMA, and AAIMA levels in both groups (Table 2).

In the comparison between T0 and T2 in Group 1, serum total thiol and albumin levels were significantly lower (*P*=0.027 and *P*=0.029, respectively) and IMA levels were higher (*P*=0.008) than in other groups. In the comparison between T0 and T2 in Group 1, there was no difference in terms of serum native thiol, disulphide, SSSH, SS total SH, SH total SH, and AAIMA (*P*>0.05). In the comparison between T0 and T3 in Group 1, serum native thiol, total thiol, SH total SH, and IMA levels were statistically significantly higher (*P*<0.001, *P*<0.001, *P*=0.013, and *P*=0.007, respectively) and SSSH and SS total SH levels were lower (*P*=0.011 and *P*=0.010, respectively) than in other groups. There was no difference between T0 and T3 in Group 1 in terms of disulphide and AAIMA levels (*P*>0.05).

There was a statistically significant increase in levels of AAIMA between T0 and T2 in Group 2 (*P*=0.014). In the comparison between T0 and T2 in Group 2, there was no difference in terms of serum native thiol, total thiol, disulphide, SSSH, SS total SH, SH total SH, and IMA (*P*>0.05). In the comparison between T0 and T3 in Group 2, serum native thiol, total thiol, SH total SH, and IMA levels were statistically significantly higher (*P*<0.001) and SSSH and SS total SH levels were lower (*P*<0.001) than in other groups. There was no difference between T0 and T3 in Group 2 in terms of disulphide and AAIMA levels (*P*>0.05).

Albumin levels were similar between the groups (*P*>0.05). In T0-T1, T0-T2, and T0-T3 comparisons between each group in terms of albumin, a significant decrease in Group 1 was detected (*P*<0.001, *P*=0.029, and *P*=0.014, respectively). The change in albumin level was statistically significant in Group 2 in T0-T1 and T0-T3 comparisons (*P*<0.001 and *P*=0.013, respectively). There was no difference between the groups in terms of length of stay in the ICU (Table 1) (*P*>0.05). Duration of hospitalization was significantly lower in Group 1 than in Group 2 (Table 1) *(P*<0.001).

## DISCUSSION

To the best of our knowledge, our study is the first to investigate the effect of continuous ventilation during CPB on thiol/disulphide homeostasis and IMA. In patients who were ventilated, native thiol and total thiol values were significantly higher at the postoperative 24^th^ hour than in patients who were not ventilated. Although IMA levels were higher at the postoperative 24^th^ hour in patients who were not ventilated than in those who were, there was no difference in terms of AAIMA levels. AAIMA levels returned to start levels at the 6^th^ postoperative hour in patients who were ventilated and at the 24^th^ postoperative hour in patients who were not ventilated. Also, the duration of hospitalization was significantly shorter in patients who were ventilated than in patients who were not ventilated.

It is known that CABG surgery increases oxidative parameters and produces a strong antioxidant response^[1-3,6,19]^. Antioxidant capacity is seen as a predictive parameter in determining postoperative complications^[20]^. IMA is believed to be triggered by a decrease in blood flow. Various studies in the literature have investigated the effects of off-pump CABG surgery, different CPB membrane systems, the use of perioperative antioxidant supplements, and pulmonary functions on the oxidative system^[1,10,20]^. There are also studies investigating the effects of perioperative mechanical ventilation strategies on inflammation in CABG^[16,21,22]^. Although there are studies on oxidative parameters in CABG^[2,8]^, there are few studies on IMA^[6,19]^ and no studies on thiol/disulphide homeostasis.

Altıparmak et al.^[23]^ showed low native thiol levels in patients with coronary artery disease (CAD) with critical stenosis who underwent coronary angiography. They demonstrated lower disulphide levels in patients with stenosis than in those without stenosis and showed that the decrease in thiols was an important indicator for CAD formation. Kundi et al.^[24]^ found out that native thiol, total thiol, and disulphide levels were lower and SS total SH was higher in patients who had acute myocardial infarction. To our knowledge, our study is the first to investigate the effect of continuous ventilation during CPB on thiol/disulphide homeostasis. Similarly to Kundi et al.^[24]^, we found out that the effect on native thiol and total thiol was more prominent in Group 2. We found out that disulphide levels were low in each group, except at the postoperative 6^th^ hour in Group 2, but there was no difference between the groups in terms of disulphide levels. In our study, especially just after CPB, native thiol, total thiol, disulphide, SH total SH, and albumin levels were significantly lower in all patients, whereas SSSH and SS total SH did not change. Native thiol and total thiol levels at T3 were higher in patients who were ventilated than in patients who were not. Native thiol and total thiol levels at T2 were higher compared with the preoperative levels in the group that was ventilated. At the postoperative 24^th^ hour, thiol/disulphide homeostasis measures did not reach baseline levels, except for disulphide levels.

Decreased blood flow may induce reactive oxygen species and, consequently, they may modify the N-terminal portion of albumin causing an increased formation of IMA. IMA is helpful in establishing diagnosis in the early stages of ischemia, before necrosis develops^[19]^. The free radical binding capacity of IMA is very low. Elevation of IMA is directly associated with free radicals that form during ischemia^[25]^.

Kanko et al.^[19]^ measured IMA levels at the 30^th^ minute of cross-clamping and at the 6^th^ postoperative hour in 30 patients who underwent CABG. The highest IMA value was found in the intraoperative period and although IMA values decreased at the postoperative 6^th^ hour, they did not reach the initial level^[19]^. Thielman et al.^[6]^ investigated IMA as a diagnostic marker of myocardial injury (MI) in patients who underwent CABG. They showed that the diagnostic value of IMA was limited and IMA values were measured as high until the postoperative 72^nd^ hour^[6]^. We found out that the IMA value was the highest in the post-pump intraoperative period, as with Kanko et al.^[19]^, and that the IMA value did not reach the initial level at the postoperative 24th hour. In patients who undergo CPB in CABG, the IMA level may be misleading because the change in the albumin value is high; the AAIMA level may be more accurate. Therefore, we looked at the values of AAIMA, different from other studies, but we found no difference between the groups. In addition, the AAIMA level reached the initial level at the postoperative 6th hour in patients who were ventilated but it took until the postoperative 24^th^ hour in patients who were not ventilated.

Luyten et al.^[2]^ focused on oxidative products induced by I/R in cardiac surgery. They measured antioxidant capacity, glutathione peroxidase, and superoxide dismutase after starting CPB, 10 minutes after CPB was finished, and at the postoperative 4^th^ and 24^th^ hours in 10 patients who underwent CABG and found an increase in those levels in the intraoperative period.

Continuous lung ventilation during CPB can reduce ischemia and lung damage by reducing the drop in blood flow to the bronchial artery^[11,13-15]^. In the literature, there are studies showing less postoperative pulmonary complications, pulmonary edema, and lung damage^[3,12,13,22]^, decreased inflammation^[3,15,22]^, better postoperative pulmonary compliance and oxygenation^[12,14,21]^, and shorter extubation time^[14]^ related to ventilation strategy. Fernando et al.^[22]^ showed in their review that lung protective methods, such as ventilation with lower tidal volumes and higher PEEP, decreased postoperative pulmonary complications and inflammation; however, they concluded that strong, randomized, and controlled studies were needed. John et al.^[13]^ showed that continuous ventilation with 5 mL/kg tidal volume during CABG presented less extravascular lung water, lung damage, and a shorter extubation time than without it. We also investigated the relationship between low tidal volume lung ventilation and length of ICU and hospital stay. In our study, we performed ventilation five times per minute with 5 mL low tidal volume without PEEP and found out that there was no difference between the groups in terms of length of ICU stay, but patients who were ventilated stayed in hospital for significantly less time than patients who were not ventilated. Durukan et al.^[16]^ used a low ventilation strategy, similarly to us, and found no difference in terms of extubation time and duration of hospital stay. In studies using continuous ventilation in CPB, no change in duration of hospital stay was found^[12,15]^.

Interruption of ventilation during CABG is one of the causes of lung injury in cardiac surgery. The lungs are very sensitive to the systemic inflammatory response effect due to CABG and I/R^[3]^. Setting different respiratory parameters in CABG surgery may positively affect clinical outcomes^[15]^. It is easy to continue ventilation during total CPB in CABG. This had a positive effect on native thiol, total thiol, and AAIMA levels in the postoperative period, and decreased the duration of hospital stay.

### Study Limitations

There are some limitations in this study. This was a single-center study. No cost measurement was performed. No inflammatory markers were measured. Postoperative pulmonary complications were not recorded. Long-term postoperative antioxidant status was not studied, and the postoperative extubation time was not recorded.

## CONCLUSION

CABG causes I/R damage and an increase in oxidation parameters that will affect the heart, lungs, and all cells. Continuous ventilation during CBP caused an increase in levels of native and total thiols at the postoperative 24th hour, early return of AAIMA levels to their initial preoperative levels, and short hospital stay. Accordingly, continuous ventilation can reduce the negative effects of CPB on the myocardium. There is a need for further studies in which different ventilation strategies should be compared and patients should be followed up for longer periods.

**Table:** 

Author's roles & responsibilities	
SEO	Substantial contributions to the conception or design of the work; or the acquisition, analysis, or interpretation of data for the work; drafting the work or revising it critically for important intellectual content; agreement to be accountable for all aspects of the work in ensuring that questions related to the accuracy or integrity of any part of the work are appropriately investigated and resolved; final approval of the version to be published
KKO	Substantial contributions to the conception or design of the work; or the acquisition, analysis, or interpretation of data for the work; drafting the work or revising it critically for important intellectual content; agreement to be accountable for all aspects of the work in ensuring that questions related to the accuracy or integrity of any part of the work are appropriately investigated and resolved; final approval of the version to be published
YU	Substantial contributions to the conception or design of the work; or the acquisition, analysis, or interpretation of data for the work; final approval of the version to be published
DK	Drafting the work or revising it critically for important intellectual content; final approval of the version to be published
BO	Drafting the work or revising it critically for important intellectual content; final approval of the version to be published
BB	Substantial contributions to the conception or design of the work; or the acquisition, analysis, or interpretation of data for the work; final approval of the version to be published
OE	Substantial contributions to the conception or design of the work; or the acquisition, analysis, or interpretation of data for the work; final approval of the version to be published
SY	Drafting the work or revising it critically for important intellectual content; final approval of the version to be published
